# On the Presence of Fibrils of Soft Tissue in the Dentinal Tubes

**Published:** 1857-06

**Authors:** John Tomes

**Affiliations:** Surgeon-Dentist to the Middlesex Hospital, Cavendish Square


### ON THE PRESENCE OF FIBRILS OF SOFT TISSUE IN THE DENTINAL TUBES·

By John Tomes, F. R. S., Surgeon-Dentist to the Middlesex Hospital.

The dental tissues, as parts of the human system, have received their full share of attention from anatomists. Papers have from time to time appeared upon this subject, each observer confirming or correcting the views of his predecessor, or adding new facts to those already recorded, until this field of investigation seemed fairly exhausted, at all events of new matter.

Histologists, I think, now agree that dentine is made up of series of tubes, which radiate from one or more cavities, situated within the interior of the tooth. In their way outwards the tubes branch freely, and connect themselves through their branches with each other; thereby establishing a network of communications throughout the whole substance of the dentine. The tubes on the one hand, after running their course, become lost in the anastomosing branches near the outer surface of what has been termed a dentinal system, on the other, terminate by open mouths on the inner surface of the system,* or pulp-cavity. This cavity being occupied by an organ rich in blood-vessels, has led to the opinion generally entertained, that the tubes are canals for the conveyance of nutritive fluid.

M. Kolliker† states, "During life the (dentinal) canals contain a clear fluid, and can not therefore be readily detected in recent preparations."

In sections which have been dried, the tubes become very distinct, and we may sometimes, on adding a colored fluid to the preparation when under the microscope, observe the tubes becoming gradually filled.

The foregoing conditions of the dentinal tubes are so easily demonstrated, and appeared to indicate so satisfactorily the offices of these canals, that the subject was regarded as one which had been fully investigated. There are, however, certain physiological conditions observable in teeth, when forming part of the living body, which the recorded knowledge of the histological characters of dentine fails to explain.

If, for instance, a portion of enamel be accidentally broken from the surface of a tooth, so that the dentine becomes exposed, the surface of the latter will be highly sensitive to any variation of temperature from that of the mouth, or to the contact of foreign bodies, even pressure from the tongue giving pain. The degree of pain is not, however, increased by increasing the pressure. Then, again, in operating upon the teeth for the removal of carious dentine, it is almost invariably found that the dentine immediately below the enamel is much more sensitive than that situated deeper in the tooth.

If the pulp of a tooth be destroyed, either by an instrument or by an escharotic, the sensitiveness of the whole of the dentine is immediately lost, no pain being afterwards experienced when it is cut either near the enamel or the pulp-cavity. The

---

* "On the Structure of the Dental Tissue of the Order Rodentia," by John Tomes, Philosophical Transactions, Part 2, 1850.

† Manual of Human Histology, by A. Kolliker. Translated and edited by George Busk, F. R. S., and Thomas Huxley, F. R. S. Vol. ii. page 41.

teeth of young subjects are much more sensitive than those of older people, and this is more especially the case when they are attacked by caries.

The dentine of teeth which are rapidly decaying is much more sensitive than that of teeth in which the destruction progresses more slowly. The former condition is indicated by the light color of the decomposing part, together with the extent of tissue involved; the latter by the deep brown color, and the comparative hardness of the affected dentine. In certain cases of caries, the softened tissue appears to be extremely sensitive, so that the patient can scarcely bear its removal; but when the instrument reaches the comparatively healthy dentine, the pain, although present, is much less severe.

In any case, however, the dentine loses its power of feeling pain if the pulp be destroyed; but if, after the destruction, the pulp-cavity be perfectly filled with gold, the tooth, in cases suitable for such an operation, may retain its color and usefulness for a considerable period. The dentine will not, however, recover its sensitiveness.

These several conditions indicate sufficiently clearly that the sensitiveness of the dentine is dependent upon its connexion with the pulp of the tooth, and that it has no inherent sensibility in its own hard tissue; although the tissue may remain for a considerable period without any manifest change, if the root of the tooth be healthy, and the dentine be protected from the influence of the fluids of the mouth.

After a portion of dentine has been for some time exposed, or if the exposure be brought about gradually by the slow wearing away of the enamel, that acute sensitiveness which has been described is not then found to exist. In parts which have been subject to the foregoing conditions, it will on examination be found that the dentinal tubes, the peripheral extremities of which have been exposed, are more or less obliterated in some part of their course between the surface and the pulp cavity.

On reviewing the various circumstances under which dentine evinces sensibility, and those under which that sensibility is lost, it is difficult to avoid the conclusion, that the dentinal tubes are in some way the medium through which sensation is distributed through the substance of the tissue. But if the sole office of the tubes be the conveyance of nutrient fluid derived from the pulp, the difficulty of accounting for the sensitiveness of the dentine remains, inasmuch as we have no instance of sensation being manifested in a fluid. We might seem to get out of the difficulty by assuming that the dentinal tubes are constantly filled by fluid, and that pressure made upon the fluid at the exposed ends of the tube is felt by the pulp at their inner extremities. This assumption does not, however, account for all the circumstances of the case, failing altogether to explain the greater sensibility of the dentine at one part of the tooth than at another.

The want of accordance between the views usually entertained upon the structure of dentine and the physiological conditions manifested by that tissue when in connexion with the body, has wholly arisen from assuming that the dentinal tubes are solely for the conveyance of fluid, and that they are otherwise empty. With the hope of gaining some further knowledge upon this point, I commenced a series of observations, the results of which it is the main purpose of this paper to communicate. When these investigations were commenced, I had but little expectation of finding that one of the most important parts in dental structure had been overlooked, namely, that each dentinal tube is permanently tenanted by a soft fibril, which, after passing from the pulp into the tubes, follows their ramifications.

With proper care in manipulating, nothing is more easy than to demonstrate the existence of the dentinal fibrils, in any tooth which has been recently extracted. If a thin section be made in the plane of the direction of the tubes, and then placed in dilute hydrochloric acid until the whole or a greater part of the lime is removed, and the section be afterwards torn in a direction transverse to that of the tubes, many of the fibrils will be seen projecting from the torn edges. It is desirable, in repeating the experiment, to place the decalcified section upon a slide before tearing, as in moving it from the surface upon which it has been torn, some of the longer fibrils may be folded back upon the body of the specimen and thus become obscured. Where the separation between the torn surfaces has been but slight, we may often see a fibril, unbroken, stretching across from the separated orifices of the tube to which it belongs.

It is not necessary, however, to decalcify dentine in order to show the fibrils. If a similar section to that already described be divided with the edge of a knife, many of these delicate organs will be seen, but they are usually broken off much shorter, many of them scarcely projecting beyond the orifices of the tubes. Again if a minute portion of dentine be cut with a sharp knife from the surface produced by fracturing a perfectly fresh tooth, the same appearances will be seen, but not with the same certainty and distinctness as in the previous examples.

In order to demonstrate the connexion of the fibrils with the pulp, fine sections should be made with a sharp knife from the edge of the pulp cavity. In this manner I obtained the specimen from which Mr. De Morgan has been kind enough to draw the accompanying illustration, showing the fibrils stretching from the pulp to the displaced dentine, and some of them passing out on the other side of the fragment. That the fibrils proceed from the pulp may be seen by carefully fracturing a fresh tooth with as little displacement of the fractured parts as possible; and then, by slowly removing the pulp from its place in the tooth, we shall be enabled to examine the fibrils which have been drawn out from the tubes. By this procedure, some of the fibrils will be withdrawn from their normal position in the dentine in the greater part of their length, a few of them retaining short lengths of their branches, but sufficient to show that they have come from the branches of the detinal tubes.

If a carious tooth be selected in which the diseased part is of a deep brown color and of tolerably firm consistence (conditions indicating that the disease has been slow in progress), it will be seen on making a transverse section of the tubes in the affected part, that the fibrils have been consolidated and their outline lost, the circumference of the tube alone being distinguishable. Indeed, the tubes, when in this state and seen in this view, have the appearance of solid rods. But if the section be made in a plane with the tubes, we shall be enabled to trace the calcified fibrils. They appear to have a greater power of resisting decomposition than the surrounding dentine, and hence preserve their rigidity. Some will project from the edge of the specimen, while others may be seen broken within the tubes, and more or less displaced. Were they made of glass the fracture could not be more abrupt and defined, or their outline more distinct. I have on a previous occasion described a zone of consolidation limiting caries,* but I was at that time ignorant of

* Lecture on Dental Physiology and Surgery, by John Tomes. Published by Parker, West Strand.

the existence of the tube-fibrils, otherwise I should have more fully understood its import.

Prof. KÖLLIKER, in his account of the developmənt of dentine, describes and figures processes extending from the peripheral cells of the dentinal pulp in developing teeth,* but he does not recognize the tube-fibril; indeed he, as before cited, describes the tubes as filled with fluid. M. LENT, in a paper published last year, gives a similar description to that of M. KÖLLIKER, but says that the cell-fibres are best seen in teeth which are but little advanced in development.† Mr. HUXLEY states that in a solitary instance he observed a fibre pass a short distance into the dentine.‡

Both M. KÖLLIKER and M. LENT regard the process which they observed extending from the peripheral cells of the pulp in forming teeth, as organisms for the development of the dentinal tubes. The latter author, near the conclusion of his article on the development of dentine, states, *the processes of the cells are the dentinal tubes* He observes further on, that the fact first observed by MULLER, and then by KÖLLIKER, that the dentinal tubules possess separate walls, which can readily be isolated, is explained by the history of the development; the wall of the dentinal canal is identical with the cellular membrane of the ivory cell.

I do not propose entering upon the subject of dentinal development in the present communication, but shall confine myself to showing that the dentinal tubes are in the normal condition occupied by fibrils of soft tissue. The above extracts from M. Lent's paper have been made in order to show that he has not recognized the existence of permanent tube contents, although he has probably seen the fibrils themselves.

The nature and office of the dentinal fibrils remain for consideration. If a fibril be examined in its natural condition, by the aid of an eighth of an inch object-glass, it will be found to consist of an almost structureless tissue, transparent, and of a comparatively low refractive power. In glycerine the fibrils are scarcely visible. At present it admits of doubt whether they are tubular or solid. In some cases there is an

---

* *Loc. cit.*

† Zeitschrift fur wissenschaftliche zoolegie, herausgegeben von C. T. SIEBOLD und KOLLIKER, Sechster Band, 1855, p. 121.

‡ On the Development of the Teeth, and on the Nature and Imports of Nasmyth's "persistent capsule," by Thomas Huxley, F. R. S., Quarterly Journal of Microscopical Science, No. 3, 1853.

appearance of tubularity; but being cylindrical, this may be a mere optical effect. When accidentally stretched between two fragments of dentine, the diameter of the fibril becomes much diminished, and when broken across, a minute globule of transparent but dense fluid may sometimes be seen at the broken end, gathered into a more or less spherical form. These appearances may be explained by assuming that the fibril consists of a sheath containing a semi-fluid matter, similar to the white fibrillæ of nerves; but whether such a conclusion can be justified, admits of doubt. The manner in which the dentinal fibrillæ terminate in the pulp, I am at present unable to decide. In favorable specimens, they may be traced a short distance into the pulp, but whether they are terminated by cells, or in any way connect themselves with nerves, I am unable to determine. The dimensions of the fibrils are the same as those of the interior of the dentinal tubes.

The conditions under which sensation is manifested in dentine have been already stated, together with those under which it is lost, and the difficulty of accounting for these phenomena has been pointed out. The recognition of the fibrils of dentine will, however, I think, remove the difficulty, and enable the physiologist to explain why under certain circumstances that tissue is susceptible of pain, while under other conditions the sensitiveness is lost.

That the dentine owes its sensation to the presence of the dentinal fibrils can not, I think, be readily doubted, seeing that if their connection with the pulp be cut off by the destruction of the latter, all sensation is at once lost. It is by no means necessary to assume that the dentinal fibrils are actual nerves, before allowing them the power of communicating sensation. Many animals are endowed with sensation which yet possess no demonstrable nervous system; and we may find many points in the human body highly sensitive without our being able to demonstrate nerves in such numbers as would account for the pain uniformly experienced from the puncture of a needle, upon the supposition that the needle had in each case wounded a nerve. Additional evidence in favor of the view that the fibrils possess sensation may be obtained by examining their condition in diseased teeth, and the conditions attendant upon the disease. In those cases in which the fibrils are consolidated in the manner already described, there is perfect absence of pain when the part is removed, but so soon as the instrument reaches the healthy dentine, more or less inconvenience is felt. If, on the other hand, there is no consolidation of the fibrils, but the pulp is yet living, the operation of removing the carious part is productive of pain, even from the commencement; indeed, pressure upon the surface of the softened tissue gives rise to discomfort. If in such cases the softened dentine be examined, fibrils may here and there be found but little altered from their natural appearance.

The greater degree of sensitiveness observable in the dentine immediately below the enamel, that is, at the point of ultimate distribution of the dentinal tubes, and consequently of the fibrils, may be fully accounted for on the supposition that the latter are organs of sensation, just as in nerves of sensation the point of greatest sensibility is that of their ultimate distribution.

The recognition of the dentinal fibrils must lead to a modification of the opinions hitherto entertained as regards the office of the tubes, namely, that they are for the circulation of fluids only. The presence of soft tissue would not, however, hinder the slow passage of fluids; and that fluids do pass through or by the side of the fibrils is rendered probable by the fact, that they are capable of undergoing change at the parts farthest removed from the pulp. When the fibrils become calcified near the surface of the dentine, the hardening material must have been derived from the pulp when the consolidation has taken place in the crown of the tooth.

The foregoing observations will, I think, warrant the conclusion, that the dentinal fibrils are subservient to sensation in the dentine, and are the channels by which nutrition is carried to that tissue.

Further evidence may be adduced in favor of the latter opinion. I have already observed that dentine may remain for a time apparently unaltered if the pulp be destroyed and the cavity filled with gold. After a while many teeth so circumstanced become loose, and when removed it is found that a considerable portion of the dentine has been removed by absorption; a state of things in some respects similar to that which accompanies the loss of teeth in old people. Here we find that, although the pulp may be living, the tubes of the root of the tooth have become consolidated, and the part rendered translucent. Teeth so circumstanced will on examination exhibit loss of dentine. A similar condition may be found in teeth the crowns of which have been lost; the roots are then diminished by absorption. In each of the instances adduced, the teeth may, however, be retained for a lengthened period in the jaw, but such persistence is always accompanied by the deposition of cementum to an unusual extent upon the roots. These phenomena have been brought forward to show that the presence of the dentinal fibrils in a state of integrity is necessary to the normal condition of the tooth; that if from any cause they are consolidated or destroyed, nature will coat over the root with cementum, and often to an extent amounting to disease, or will set up a process for its removal. The dentine will be diminished by absorption, the root will be thrown up on the surface of the gum, or the socket will disappear, and the tooth, by the one or other process, or by a combination of each, will be cast off as an organ no longer fitted for a place in the living body.

### ADDENDUM.

Since the preceding communication has been in the possession of the Royal Society, the head of a marsupial animal which had been preserved in spirit was placed at my disposal, the teeth of which were in a condition favorable for showing the dentinal fibrils, should such be found to exist.

A paper upon the structure of the dental tissues af Marsupiata will be found in the Philosophical Transactions, Part II. 1849, in which the continuation of the dentinal tubes into the enamel is described and figured; together with those minor differences of structure which are peculiar to the several divisions of this order of Mammalia.

After the discovery of the dentinal fibrils, the examination of a favorable specimen of enamel so peculiarly constituted became a matter of considerable interest, in order to ascertain whether the soft tissue which occupies the dentinal tubes is continued into those of the enamel. I am indebted to my friend Professor Quekett for the jaws of *Halmaturus* ——, a member of a genus in which the majority of the dentinal tubes situated in the crown of the tooth are continued into the enamel, and pass to within a short distance of the external surface of that tissue. Thin sections were made both of the incisor and molar teeth by the usual process of grinding.

These were treated with dilute hydrochloric acid, and were examined at short intervals after their immersion in the solvent fluid. The acid acted upon the enamel with great rapidity; and in the course of a few minutes the edge corresponding to the outer surface of the tooth disappeared, leaving in its place a series of fine flexible filaments. More prolonged action of the acid led to a further loss from the surface of the enamel, and also to the solution of the part in contact with the dentine. In this case the fibrils were seen proceeding from the extremities of the dentinal tubes across the space which had been occupied by the enamel, from thence they were continued through that portion of the latter structure which yet remained undissolved, and ultimately formed a delicate fringe floating freely in the fluid by which the preparation was surrounded. If a section presenting the above conditions be again placed in acid, the whole of the enamel will be dissolved, leaving the dentine in those parts which have been invested with enamel, bordered by a thick fringe of long delicate fibrils, each one being continued from the peripheral extremity of a dentinal tube. In the dentine the fibrils occupying the tubes were as readily detected as in the human tooth, and presented the same general appearances and relations.

The facility with which the fibrils were demonstrated in the enamel of the teeth of the Kangaroo, induced me to select for examination specimens of human teeth, in which the dentinal tubes are continued for a short distance into the enamel. Under similar treatment, similar results were obtained. Wherever the dentinal tubes could be traced into the enamel, the presence of the contained fibrils could be demonstrated by the aid of hydrochloric acid.

CAVENDISH SQUARE, June 17th, 1856.

